# The effects of short-term, progressive exercise training on disease activity in smouldering multiple myeloma and monoclonal gammopathy of undetermined significance: a single-arm pilot study

**DOI:** 10.1186/s12885-024-11817-6

**Published:** 2024-02-05

**Authors:** A Emery, S Moore, J Crowe, J Murray, O Peacock, D Thompson, F Betts, S Rapps, L Ross, D Rothschild-Rodriguez, A Arana Echarri, R Davies, R Lewis, DX Augustine, A Whiteway, Z Afzal, JLJ Heaney, MT Drayson, JE Turner, JP Campbell

**Affiliations:** 1 Department for Health, University of Bath, Bath, UK; 2 Department for Haematology, Royal United Hospitals Bath NHS Foundation Trust, Bath, UK; 3 School of Biological Sciences, University of Southampton, Southampton, UK; 4 Department for Physiotherapy, Royal United Hospitals Bath NHS Foundation Trust, Bath, UK; 5 Department for Cardiology, Royal United Hospitals Bath NHS Foundation Trust, Bath, UK; 6 Department for Haematology, North Bristol NHS Trust, Bristol, UK; 7 Clinical Immunology Service, Institute of Immunity and Immunotherapy, College of Medical and Dental Sciences, University of Birmingham, Birmingham, UK; 8 School of Sport, Exercise and Rehabilitation Sciences, College of Life and Environmental Sciences, University of Birmingham, Edgbaston, Birmingham, UK; 9 School of Medical and Health Sciences, Edith Cowan University, WA Joondalup, Australia

**Keywords:** Exercise oncology, Aerobic exercise, Resistance exercise, Anti-cancer mechanisms, Cancer precursors, Cancer risk

## Abstract

**Background:**

High levels of physical activity are associated with reduced risk of the blood cancer multiple myeloma (MM). MM is preceded by the asymptomatic stages of monoclonal gammopathy of undetermined significance (MGUS) and smouldering multiple myeloma (SMM) which are clinically managed by watchful waiting. A case study (*N* = 1) of a former elite athlete aged 44 years previously indicated that a multi-modal exercise programme reversed SMM disease activity. To build from this prior case study, the present pilot study firstly examined if short-term exercise training was feasible and safe for a group of MGUS and SMM patients, and secondly investigated the effects on MGUS/SMM disease activity.

**Methods:**

In this single-arm pilot study, *N* = 20 participants diagnosed with MGUS or SMM were allocated to receive a 16-week progressive exercise programme. Primary outcome measures were feasibility and safety. Secondary outcomes were pre- to post-exercise training changes to blood biomarkers of MGUS and SMM disease activity– monoclonal (M)-protein and free light chains (FLC)– plus cardiorespiratory and functional fitness, body composition, quality of life, blood immunophenotype, and blood biomarkers of inflammation.

**Results:**

Fifteen (3 MGUS and 12 SMM) participants completed the exercise programme. Adherence was 91 ± 11%. Compliance was 75 ± 25% overall, with a notable decline in compliance at intensities > 70% V̇O_2PEAK_. There were no serious adverse events. There were no changes to M-protein (0.0 ± 1.0 g/L, *P* =.903), involved FLC (+ 1.8 ± 16.8 mg/L, *P* =.839), or FLC difference (+ 0.2 ± 15.6 mg/L, *P* =.946) from pre- to post-exercise training. There were pre- to post-exercise training improvements to diastolic blood pressure (− 3 ± 5 mmHg, *P* =.033), sit-to-stand test performance (+ 5 ± 5 repetitions, *P* =.002), and energy/fatigue scores (+ 10 ± 15%, *P* =.026). Other secondary outcomes were unchanged.

**Conclusions:**

A 16-week progressive exercise programme was feasible and safe, but did not reverse MGUS/SMM disease activity, contrasting a prior case study showing that five years of exercise training reversed SMM in a 44-year-old former athlete. Longer exercise interventions should be explored in a group of MGUS/SMM patients, with measurements of disease biomarkers, along with rates of disease progression (i.e., MGUS/SMM to MM).

**Registration:**

https://www.isrctn.com/ISRCTN65527208 (14/05/2018).

**Supplementary Information:**

The online version contains supplementary material available at 10.1186/s12885-024-11817-6.

## Background

Monoclonal gammopathy of undetermined significance (MGUS) and smouldering multiple myeloma (SMM) are asymptomatic precursors to the blood cancer multiple myeloma (MM) [1], and typically arise in older adults, affecting > 4% of adults aged > 50 years in the general population [2, 3]. The differential diagnosis of MGUS *vs.* SMM is defined by < 10% or ≥ 10% cancerous bone marrow plasma cells, and monoclonal (M)-protein in blood < 30 g/L or ≥ 30 g/L, respectively [4]. A diagnosis of MM is confirmed with MM-associated symptoms and/or a MM-defining event [4]. The overall risk of disease progression to MM ranges from 1 to 10% per year for MGUS and SMM, respectively [5, 6]. Despite the risk of progression to MM, treatment with conventional anti-cancer therapies is not advocated for MGUS and SMM [7]. Instead, MGUS and SMM are managed by ‘watchful waiting’ with routine measurements of blood biomarkers: M-protein and free light chains (FLC) [8].

Considering the relatively non-invasive and accessible nature of disease biomarkers in blood and the absence of therapeutic intervention, MGUS and SMM represent a relevant clinical model to explore the isolated effects of exercise training on early cancer disease activity in humans. Only one study to date– a case study of one patient– has investigated the effects of exercise training on SMM disease activity [9]. Across a two-year period of moderate training (10–20 h/week) following SMM diagnosis, M-protein decreased from 32.9 g/L to 25.3 g/L (− 23%) [9]. Subsequently, a three-year period of supervised multi-modal training was performed for 12–20 h/week, and M-protein decreased further from 25.3 g/L to 18.4 g/L (− 27%) [9]. The patient was aged 44 years– which is relatively young in the context of MGUS and SMM [3]– and was a former elite handball athlete with a 20-year training history at high volumes (> 30 h/week) prior to diagnosis of SMM [9]. Thus, the findings of this case study need validating in a demographically broader group of MGUS and SMM patients.

As with many cancers, the risk of developing MM is reduced among people reporting the highest levels of physical activity in the population [10], however, the mechanisms by which physical activity reduces symptomatic cancer risk remain to be elucidated. Importantly, evidence from epidemiology studies demonstrates that the earliest manifestations of cancer– so called cancer ‘precursors’– do not appear to be prevented in people reporting high levels of physical activity [11, 12], suggesting that the anti-cancer mechanism(s) of physical activity are induced after early neoplasia (e.g., MGUS) has arisen [13]. This idea is reinforced by the previously summarised case study where the patient was an elite athlete for 16 years (1985–2001) and completed > 30 h/week training for four years (2001–2004) prior to a diagnosis of SMM in 2005 [9].

The anti-cancer mechanism(s) of physical activity, which result in delayed tumour outgrowth, remain unknown. It is widely predicted that physical activity prevents cancer outgrowth either by suppressing endogenous systemic factors (e.g., inflammation) that induce cell division/damage/proliferation, or enhancing immune competency, as reviewed recently elsewhere [13]. For example, one prominent hypothesis proposes that acute bouts of exercise augment cancer immunosurveillance and elimination in peripheral tissuesby natural killer (NK) cells or T cells [14–16]. In the case of MGUS/SMM, this hypothetical exercise-induced immunosurveillance response would take place in the bone marrow where the tumour bulk comprised of long-lived neoplastic plasma cells resides [17]. This immunosurveillance response to acute exercise is intensity-dependent, with a larger-magnitude mobilisation of leukocytes in response to higher/vigorous intensity exercise compared to lower/moderate intensity exercise [16, 18].

It is thus of interest to investigate how short-term exercise training–and particularly vigorous intensity aerobic exercise– affects disease activity in MGUS/SMM. To achieve this outcome, the safety and feasibility of vigorous intensity exercise training for patients with MGUS/SMM should be confirmed. Secondly, it is important to determine whether short-term exercise training alters disease activity, as indicated by a prior case study which included high-intensity exercise modalities [9]. 

Therefore, the primary aim of this pilot study was to assess the feasibility and safety of aerobic exercise progressing from moderate intensity to vigorous intensity in MGUS/SMM. Specifically, to assess the proportion of exercise sessions attended (i.e., adherence) and the proportion of exercise sessions completed as prescribed (i.e., compliance). The secondary aim was to measure pre- to post-exercise training changes to blood biomarkers of MGUS and SMM disease activity. In addition to assessing the feasibility, safety, and efficacy of vigorous intensity exercise in MGUS and SMM, a separate objective of this pilot study was to evaluate the effects of exercise training on fitness and wellbeing outcomes in MGUS and SMM. Physiological and quality of life benefits of exercise training are apparent in MM [19, 20] but have not yet been investigated in MGUS and SMM. As such, resistance, balance, and flexibility training were included in the intervention, in line with exercise recommendations for older adults [21]. Finally, we also assessed the effects of the exercise programme on body composition, blood immunophenotype, and biomarkers of inflammation.

## Methods

### Public and patient involvement

Collaborative discussions with patient contributors during the development and ethical approval stages of this study shaped its design. Patients appreciated the relevance of the exercise intervention, stating: “the NHS [National Health Service of the United Kingdom] is under pressure and so it is important that everyone tries to look after their own health by good lifestyle choices”. When asked about practicalities, patients indicated a preference for early morning or evening exercise sessions to allow those in employment to take part. Patients agreed that the hospital was an appropriate location for exercise sessions: “at least [they] would be at the hospital if anything goes wrong”. Patients were familiar with resistance bands, and these were adopted in the exercise intervention, and patients welcomed the use of fitness trackers to monitor exercise training, citing prior positive experiences using similar devices: “I like gadgets, so this would be great”. When asked about using a treadmill, patients were “happy to walk but not run”.

### Study design

This single-arm pilot study included measurements made in week 0 (pre-exercise training) and week 17 (post-exercise training), separated by a 16-week progressive exercise programme. The study was conducted predominantly at the Royal United Hospitals Bath NHS Foundation Trust, with two measurement visits at the University of Bath. Participants received usual clinical care throughout the study. The protocol was approved by the NHS Research Ethics Committee (reference 18/LO/1034, 20th July 2018) and University of Bath Research Ethics Approval Committee for Health (reference EP 17/18 210, 29th August 2018). The study was prospectively registered (ISRCTN 65,527,208, 14th May 2018). The pilot trial protocol is available online: https://www.isrctn.com/ISRCTN65527208. It should be noted that the trial commenced with a randomised-controlled design (*N* = 20 exercise arm *vs. N* = 20 control arm), but it was concluded at an early stage to be infeasible in the time available, due to low recruitment numbers in the large yet relatively sparsely populated catchment area of the Royal United Hospitals Bath NHS Foundation Trust. An alternative single-arm trial design (ethics amendment 19th November 2018) was deemed appropriate due to the primary interest in assessing the feasibility of the intervention, and secondary pre-post analyses of disease biomarkers in a patient group where spontaneous disease reversal is uncommon. Furthermore, a participant identification centre– North Bristol NHS Trust– was added to increase recruitment (ethics amendment 21st June 2019). 

### Recruitment

**Fig. 1:**
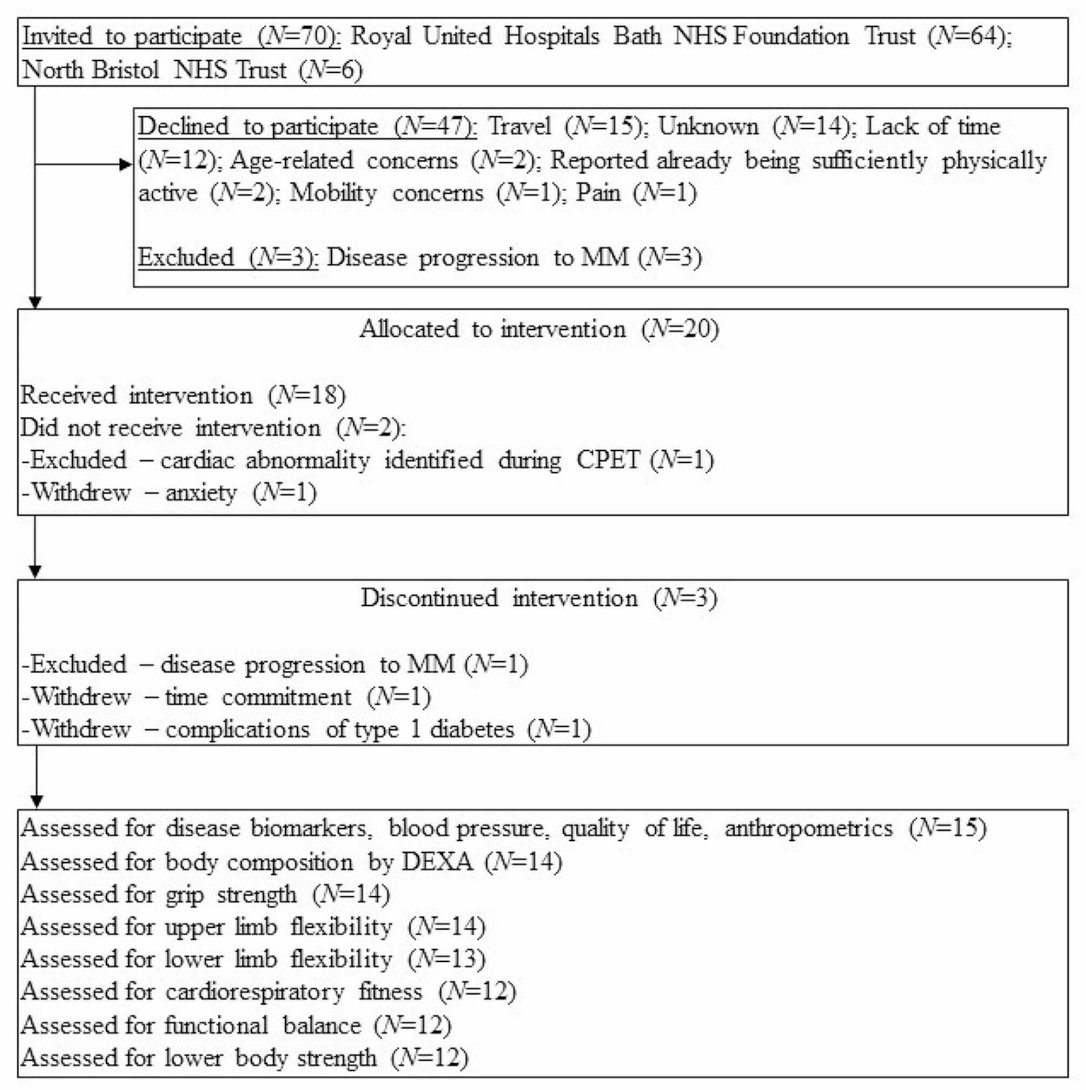
CONSORT diagram showing flow of participants through the pilot trial Missing data: DEXA (*N* = 1 participant declined radiation exposure); grip strength and upper limb flexibility (*N* = 1 did not attend for follow-up fitness measurements); lower limb flexibility (*N* = 1 did not attend for follow-up fitness measurements, *N* = 1 unable to perform due to lower limb pain at follow-up assessment); cardiorespiratory fitness, functional balance, lower body strength (*N* = 1 did not attend for follow-up fitness measurements, *N* = 2 unable to perform due to lower limb pain at follow-up assessment). MM = Multiple myeloma; CPET = Cardiopulmonary exercise test; DEXA = Dual-energy x-ray absorptiometry

Recruitment prioritised patients with the most advanced disease and highest risk of progression to MM according to risk stratification models for MGUS/SMM [7, 22]. As such, the following priority was implemented: high-risk SMM > intermediate-risk SMM > low-risk SMM > high-risk MGUS > high-intermediate-risk MGUS > low-intermediate-risk MGUS > low-risk MGUS. Recruitment materials were posted to potentially-eligible patients identified via screening of medical records. A follow-up phone call was made to ascertain willingness to be involved in the trial. Uptake was 29% and reasons for declining to take-part were recorded (Fig. 1). The most commonly cited barriers to participating were ‘lack of time’ and ‘travel burden’. Patient enrolment started in September 2018 and was completed in November 2019. Trial measurements commenced in January 2019 and were completed in March 2020.

### Participants

Twenty individuals diagnosed with SMM (*N* = 15) or MGUS (*N* = 5) according to International Myeloma Working Group guidelines [4] and aged > 18 years were recruited from the Royal United Hospitals Bath NHS Foundation Trust (*N* = 19) and North Bristol NHS Trust (*N* = 1). Participants were screened by a haematologist according to the exclusion criteria: (1) Eastern Co-operative Oncology Group (ECOG) performance status > 1, (2) pregnancy, (3) contraindication to exercise identified by physical activity readiness questionnaire, (4) cognitive impairment deemed a risk for participation in the study, (5) inability to understand explanations or provide informed consent and (6) any condition or behaviour that would introduce personal risk or bias into the study. All participants provided written informed consent in the presence of a haematologist.

### Measurements

#### Blood measurements

Venous blood samples (50 mL) were collected pre-exercise training (week 0) and post-exercise training (week 17) between 08:30 and 10:30 following a ≥ 10-h fast without food or caffeine, ≥ 24 h without alcohol intake, ≥ 20 h without strenuous exercise, and 25 min seated rest.

#### Blood processing

Whole blood collected in untreated syringes was dispensed into K3EDTA-coated tubes (1.6 mg/mL of blood, Sarstedt, Numbrect, Germany) and serum-separator tubes containing a clotting activator (Sarstedt, Numbrect, Germany). Whole blood mixed with K3EDTA was centrifuged immediately at 3000 × g for 10 min at 4 °C. Plasma was extracted and stored immediately on dry ice prior to freezing at − 80 °C until analysis. Whole blood in serum separator tubes was allowed to clot for 15 min at room temperature prior to centrifugation at 3000 × g for 10 min at 4 °C. Serum was extracted and stored immediately on dry ice prior to freezing at − 80 °C until analysis.

Whole blood collected in syringes treated with sodium heparin (5 IU/mL of blood, Wockhardt, Wrexham, UK) was maintained at room temperature for isolation of peripheral blood mononuclear cells (PBMCs) via density gradient centrifugation within two hours of blood collection. Briefly, whole blood was diluted two-fold in Roswell Park Memorial Institute (RPMI)-1640 media (Sigma-Aldrich, Dorset, UK), layered over Ficoll-Paque (Sigma-Aldrich, Dorset, UK) and centrifuged at 500 × g for 25 min. PBMCs were aspirated, washed twice in RPMI-1640 (400 × g for 10 min, then 200 × g for 10 min), and quantified using a haemocytometer. After quantification, PBMCs were centrifuged at 300 × g for 7 min and cryopreserved in RPMI-1640 containing 20% foetal calf serum (FCS) and 10% dimethyl sulfoxide (DMSO) at − 1 °C/minute using a freezing container (Nalgene Mr Frosty, ThermoFisher Scientific, Paisley, UK), prior to freezing at − 150 °C until analysis by flow cytometry.

#### Flow cytometry

Cryopreserved PBMCs were thawed rapidly at 37 °C, washed in RPMI-1640 containing 10% FCS and 1% Penicillin-Streptomycin, and quantified using a haemocytometer. After resting overnight at 37 °C and 5% CO_2_ for 14–16 h, quantification was repeated and PBMCs were washed and resuspended in phosphate-buffered saline (PBS). Viability was determined using a fixable viability stain (FVS620, BD Biosciences, Franklin Lakes, NJ, USA). After blocking non-specific antibody binding (Human Fc Block, BD Biosciences, Franklin Lakes, NJ, USA), 0.5 × 10^6^ cells in 100 µL buffer (PBS containing 2% FCS and 0.4% EDTA) were stained with titrated volumes of anti-CD3 (AF700, IgG1, clone UCHT1), anti-CD4 (PE-Cy7, IgG1, clone SK3), anti-CD8 (APC-H7, IgG1, clone SK1), anti-CD45RA (BB515, IgG2b, clone HI100), anti-CD27 (BV480, IgG1, clone L128), anti-CD57 (APC, IgM, clone NK-1), anti-PD1 (BB700, IgG1, clone EH12.1), anti-CTLA4 (PE, IgG2a, clone BNI3), and anti-KLRG1 (BV421, IgG2a, clone 14C2A07). All antibodies were purchased from BD Biosciences (Franklin Lakes, NJ, USA) with the exception of anti-KLRG1 which was purchased from BioLegend (San Diego, CA, USA). Samples were analysed within two hours of preparation using a FACS Aria III and FACS Diva software (BD Biosciences, Franklin Lakes, NJ, USA). Cell populations were defined using Flow Jo v10.6.2 (Supplemental Fig. 1). Absolute cell counts were computed by multiplying the lymphocyte differential– obtained from K3EDTA-treated whole blood on the day of blood collection via the Coulter principle (KX-21 N Haematology Analyser, Sysmex Ltd, Milton Keynes, UK)– with the percentage of cells within the CD3^+^ gate. Cell frequencies for subsequent gated daughter subpopulations were calculated by multiplying their proportion within the appropriate parent population– derived from Flow Jo analysis– by the computed frequency of the parent population [23, 24].

#### Disease biomarker analysis

Disease biomarker analysis was performed at the Clinical Immunology Service, Birmingham, UK. Protein electrophoresis and densitometry (Interlab, Rome, Italy) were used to quantify serum M-protein concentration. Serum kappa and lambda FLC (Freelite®, The Binding Site, Birmingham, UK) and polyclonal IgG, IgA, and IgM, and C-reactive protein (CRP) (Roche, Basel, Switzerland) were measured on a Cobas® 6000 Modular (Roche Hitachi Diagnostics, Basel, Switzerland). Historical M-protein and involved FLC data were obtained from medical records for three years prior to study enrolment or since diagnosis if diagnosed within three years of enrolling onto the study.

FLC ratio was calculated as: kappa FLC (mg/L) ÷ lambda LC (mg/L) and FLC difference (mg/L) was calculated as: involved FLC (mg/L) − uninvolved FLC (mg/L). Immunoparesis describes the suppression of uninvolved, polyclonal immunoglobulins, therefore change to polyclonal IgA, IgG, and IgM is only shown in patients without an IgA, IgG, or IgM M-protein, respectively, as reported previously [25]. For example, for a participant with an IgG M-protein, change to polyclonal IgA and IgM was investigated, but not polyclonal IgG.

#### Inflammatory biomarker analysis

Plasma interleukin (IL)-1 receptor agonist (IL-1RA) (V-PLEX Human Cytokine Panel 2, Mesoscale Discovery, Maryland, USA), IL-15, IL-16, IL-17 A, vascular endothelial growth factor (VEGF) (V-PLEX Human Cytokine Panel 1, Mesoscale Discovery, Maryland, USA), IL-1β, IL-4, IL-6, IL-8, IL-10, IL-12p70, and tumour necrosis factor (TNF)-α (V-PLEX Human Proinflammatory Panel 1, Mesoscale Discovery, Maryland, USA) were measured according to manufacturer’s instructions. Plasma samples were assayed in duplicate (coefficient of variation (CV) for detectable analytes = 7.6%). Plasma sIL-6Rα was measured (Human IL-6 sR Quantikine Kit, Biotechne, Abingdon, UK) according to manufacturer’s instructions and samples analysed in duplicate (CV = 3.2%).

#### Physiological measurements

Measurements were collected in week 0 (pre-exercise training) and week 17 (post-exercise training).

#### Body composition

Body composition was estimated using a whole-body dual-energy x-ray absorptiometry (DEXA) scan, fasted, without footwear, and wearing light clothing. Participants consumed 500 mL of water and voided their bladder prior to the scan and were positioned supine (Discovery, Hologic, Bedford, UK) with feet equally spaced and arms with an even gap from the trunk. Whole-body composition analysis was performed with regions sectioned as recommended (Hologic, Bedford, UK). Body mass was measured using electronic scales and standing height was measured using a stadiometer. Waist circumference was measured at the mid-point between the lowermost point of the costal margin and uppermost point of the iliac crest at the end of an exhalation. Hip circumference was measured at the level of the greater trochanter.

#### Resting cardiovascular measurements

Systolic and diastolic blood pressure, and heart rate were measured in a seated position using an automated sphygmomanometer after 25 min of seated rest.

#### Cardiorespiratory fitness

Cardiorespiratory fitness was measured using a progressive, incline-based, multi-stage cardiopulmonary exercise test (CPET) to exhaustion performed on a treadmill (LE300CE, H/P/Cosmos, Traunstein, Germany). Walking speed was self-selected and constant (range 2.0-6.5 km/h). After a 5-min warm up at 0% gradient, the gradient increased by 3% every 3-min until volitional fatigue [26]. Heart rate was recorded continuously via electrocardiogram (ECG) (Vyntus, Vyaire Medical, Basingstoke, UK) and oxygen uptake by indirect calorimetry (Vmax Vyntus, Vyaire Medical, Basingstoke, UK). At the end of each 3-min stage, rating of perceived exertion (RPE) was reported on a 6–20 scale and blood pressure was measured using an automated sphygmomanometer. Ventilatory threshold was determined via the three-point discrimination technique (modified V-slope, ventilatory equivalents, end-tidal pressures) and peak oxygen uptake (V̇O_2PEAK_) was reported as the highest 30-second average. Maximal exertion was verified by meeting two of the following secondary criteria: (1) heart rate ≥ age-predicted maximum (220–age in years), (2) respiratory exchange ratio ≥ 1.10, (3) RPE ≥ 19, or (4) an increment in V̇O_2_ ≤ 5 mL/kg/min in response to increased gradient [ 26]. Maximal exertion was achieved in 85% of exercise tests (*N* = 1 sub-maximal at baseline, *N* = 3 sub-maximal at follow-up). Two sub-maximal tests at follow-up were attributed to participants achieving the maximum workload available on the treadmill (incline 24% at walking pace). The remaining sub-maximal tests were attributed to participant-related factors (e.g., motivation). Maximal and sub-maximal tests were included in analysis.

#### Functional fitness

Dynamic balance was measured as time to complete the 8ft up-and-go test. Lower body muscle strength was measured as the number of repetitions completed in a 30-second sit-to-stand test. Upper limb flexibility was measured using the back scratch test and lower limb flexibility was measured using the sit-and-reach test. Upper-body strength was measured using a handgrip dynamometer (Takei 5401 Grip D, Niigata City, Japan).

#### Quality of life

Quality of life was assessed via satisfaction with life score and 36-item short form (SF-36) questionnaire (v1.0). Sleep quality was quantified via Pittsburgh sleep quality index (PQSI) and fatigue via functional assessment of chronic illness therapy (FACIT)-fatigue scale.

#### Habitual physical activity

Habitual physical activity was measured prior to the first laboratory measurement visit to assess baseline physical activity level. A physical activity monitor (BodyMedia Core, BodyMedia Inc., Pittsburgh, PA, USA) was positioned over the triceps muscle of the left arm. A minimum of five days including Saturday and Sunday with ≥ 80% average daily wear time was required for inclusion in analysis [27]. Energy expenditure was estimated using proprietary algorithms (SenseWear Pro 8.0, algorithm v5.2, BodyMedia Inc., Pittsburgh, PA, USA). Non-wear time was assigned estimated basal metabolic rate [28]. Participants were defined as having a high or low physical activity level (PAL) according to a threshold of 1.75 [29].

### Exercise programme

Participants were prescribed a 16-week exercise programme comprising two supervised sessions of aerobic and resistance exercise and one home-based aerobic exercise session per week (Supplemental Fig. 2). The Consensus on Exercise Reporting Template (CERT) is included in Supplemental Table 1.

#### Supervised exercise training

Supervised sessions were performed in small groups of 2–3 participants within the hospital physiotherapy gym and were supervised by an exercise physiologist. Supervised sessions included a warm-up and cool-down. Supervised aerobic exercise was a 30-minute treadmill walk, performed as three 10-min walking bouts. Each bout involved 8-min of uphill walking at intensities prescribed from V̇O_2PEAK_ assessed pre-exercise training (“exercise interval”), followed by 2-min of active recovery at 0% incline. The exercise interval was prescribed at a moderate intensity– defined by the American College of Sports Medicine (ACSM) as 46–63% V̇O_2PEAK_ [ 30]– initially to allow safety to be evaluated prior to progressing to vigorous intensities– defined by ACSM as 64–90% V̇O_2PEAK_ [30 ]– which are thought to engage purported anti-cancer mechanisms, such as immunosurveillance by T cells and NK cells [14–16]. Specifically, the intensity of the exercise interval progressed from 40–50% V̇O_2PEAK_ (weeks 1–2), to 50–60% V̇O_2PEAK_ (weeks 3–6), to 60–70% V̇O_2PEAK_ (weeks 7–10), and finally to 70–80% V̇O_2PEAK_ (weeks 11–16) (Supplemental Fig. 2). Heart rate was continuously monitored (Polar H10, Polar, Kempele, Finland) and RPE was recorded using a 6–20 scale at the end of each 8-min exercise interval. Where heart rate did not reach the prescribed intensity target in a given 8-min exercise interval, the workload was increased for the subsequent 8-min exercise interval, as tolerated by the participant.

Following treadmill walking, supervised resistance exercises were completed for six major muscle groups (reverse fly, chest fly, core twist, squat, leg abduction, band pull-through). Resistance was applied with elastic bands (Meglio, Reading, UK). Familiarisation was performed in weeks 1–2 by performing two submaximal sets of 15 repetitions. Progression comprised two sets of: 12 repetition-maximum (weeks 3–6), then ten repetition-maximum (weeks 7–10), and finally eight repetition-maximum (weeks 11–16), where resistance was increased to constrain the maximum number of repetitions performed (Supplemental Fig. 2). A repetition-maximum assessment was performed prior to each progression, where participants were instructed to perform multiple sets of the target number of repetitions for each exercise with increasing resistance until the target could not be reached or ≤ 1 repetition in reserve was reported. To ensure continued progression, participants were instructed to work past the prescribed repetition-maximum and, if they exceeded the repetition target, additional resistance was added in the next session [31].

#### Unsupervised exercise training

Home-based exercise centred around one weekly 40-min walk at a moderate intensity (RPE 12–13/20) [30] where duration was recorded with a wrist-based fitness tracker (Polar A370, Polar, Kempele, Finland). Participants were provided with NHS balance exercises [32] and flexibility exercises to perform daily. Flexibility exercises were static stretches of the pectorals, deltoids, triceps, latissimus dorsi, adductors, gastrocnemius, quadriceps, and hamstrings.

#### Feasibility and safety measurements

Adherence to the intervention was reported as the proportion of supervised exercise sessions attended and home-based exercise sessions completed using exercise record cards and diaries, respectively. Compliance to supervised aerobic exercise was assessed using a chest-based heart rate sensor (Polar H10, Polar, Kempele, Finland) for gold-standard heart rate monitoring equivalent to ECG [33]. The mean heart rate– calculated from second-by-second heart rate measurements during each 8-min exercise interval– was calculated for each supervised exercise session and interpreted in relation to the prescribed target heart rate derived from pre-exercise training CPET. As previously reported, each intensity progression was defined as feasible if > 70% of participants completed > 75% of sessions at the prescribed intensity [34]. Compliance to supervised resistance exercise was assessed by comparing the average number of repetitions performed per exercise per session, to the target repetition-maximum prescription.

Participants were provided with a wrist-based fitness tracker (Polar A370, Polar, Kempele, Finland) for the duration of the study, which was configured with participant details (sex, age, height, body mass). Compliance to the duration of home-based walks was determined by comparing the duration recorded using the fitness tracker to the prescribed duration. Compliance to the intensity of home-based walks was monitored by comparing the RPE reported in exercise diaries to the prescribed RPE. Safety was reported as the incidence, severity, expectedness, and relatedness of adverse events categorised by haematologists according to Common Terminology Criteria for Adverse Events guidelines (v5.0).

### Statistical analysis

Feasibility outcomes were reported as percentages. Statistical analysis was performed using GraphPad Prism (version 8, GraphPad Software, California, USA) and SPSS Statistics (version 27, IBM SPSS Statistics for Windows, New York, USA) with statistical significance accepted at *P* <.05. All data are presented as median ± interquartile range (IQR). The CV between pre- and post-exercise training measurements of disease activity biomarkers were calculated for each participant (CV% = (SD between pre- and post-exercise training values ÷ mean of pre- and post-exercise training values) × 100). CV values were compared to published thresholds for physiological variation to M-protein (CV 7.8%) and involved FLC (CV 27.8%) during monitoring of stable disease [35]. Compliance to supervised aerobic exercise intensity at four prescribed training intensities were tested for normality using a Shapiro-Wilk test. Due to violation of the assumption of normal distribution, a non-parametric Friedman’s ANOVA was used to identify whether there was a statistically significant difference in compliance at increasing exercise intensities. Pre- to post-exercise training change scores were tested for normality using a Shapiro-Wilk test. Pre- to post-exercise training changes for normally distributed variables were analysed using paired-samples T tests, and non-normally distributed variables were analysed using Wilcoxon signed-rank tests. An ANCOVA was used to examine whether there was an effect of baseline physical activity level (high *vs.* low) on disease activity measured post-exercise training, with pre-exercise training disease activity included as a covariate. Effect sizes were calculated for normally distributed data only and interpreted according to guidelines; small effect *d* = 0.2, moderate effect *d* = 0.5, and large effect *d* = 0.8.

## Results

### Baseline characteristics

Twenty participants took part and fifteen participants, comprising eight males and seven females with an age range of 32 years to 74 years, of which three had MGUS and 12 had SMM, completed the study (75% retention). Reasons for withdrawing (*N* = 3) and exclusion during the trial (*N* = 2) are shown in Fig. 1. Participant characteristics and clinical details are displayed in Table 1. Results are displayed herein for *N* = 15 participants that completed the study.

**Table 1:** Baseline characteristics of trial participants

	Median	IQR
**Age** (years)	63	8
**Height** (m)	1.72	0.13
**Body mass** (kg)	80.4	31.4
**BMI** (kg/m^2^)	27.3	8.7
**M-protein** (g/L, *N* = 14)	13.4	8.6
**FLC ratio**		
Overall	4.44	12.50
Involved kappa FLC (*N* = 9)	10.25	17.56
Involved lambda FLC (*N* = 4)	0.09	0.04
Normal FLC ratio (*N* = 2)	1.01	0.01
	***N***	**Percentage**
**Sex**		
Male	8	53
Female	7	47
**Diagnosis**		
MGUS	3	20
SMM	12	80
**Isotype**		
MGUS IgG Kappa	1	7
MGUS IgG Lambda	1	7
MGUS Bi-clonal IgA Kappa	1	7
SMM IgG Kappa	5	33
SMM IgG Lambda	3	20
SMM IgA Kappa	2	13
SMM Bi-clonal IgA Lambda + IgG Lambda	1	7
SMM Kappa light chain only	1	7
**Risk stratification**		
MGUS High-intermediate risk^1^	1	7
MGUS Low-intermediate risk^1^	1	7
MGUS Low risk^1^	1	7
SMM High risk^2^	3	20
SMM Intermediate risk^2^	3	20
SMM Low risk^2^	6	40
**Immunoparesis**		
Affecting one polyclonal immunoglobulin	5	33
Affecting two polyclonal immunoglobulins	5	33
Affecting three polyclonal immunoglobulins	1	7
Immunoparesis not evident	4	27
**Ethnicity**		
White	14	93
Mixed/multiple	1	7
**Employment status**		
Employed	10	67
Retired	4	27
Unable to work	1	7
**Smoking status**		
Never-smoker	10	67
Ex-smoker	5	33
**BMI category**		
Healthy (< 25 kg/m^2^)	5	33
Overweight (25-29.9 kg/m^2^)	5	33
Obese (> 30 kg/m^2^)	5	33
**Physical activity level**		
High (PAL ≥ 1.75)	8	53
Low (PAL < 1.75)	7	47

IgG or IgA, and kappa or lambda indicate the involved, clonal immunoglobulin heavy chain and light chain, respectively, of the plasma cell neoplasia ^1^MGUS risk stratification [7]; ^2^SMM risk stratification [22]. IQR = Interquartile range; BMI = Body mass index; MGUS = Monoclonal Gammopathy of Undetermined Significance; SMM = Smouldering Multiple Myeloma; PAL = Physical activity level (measured using BodyMedia Core device)

### Intervention adherence, compliance, and safety

Attendance at supervised exercise sessions was 91 ± 11% and participation in home-based walks was 88 ± 34%. The prescribed exercise duration was achieved in 100 ± 3% of supervised treadmill walks, and the prescribed exercise intensity was achieved in 75 ± 25% of supervised treadmill walks. Analysis of aerobic exercise intensity progression revealed a decline in compliance at intensities > 70% V̇O_2PEAK_, where the proportion of compliant sessions decreased by ~ 23% compared to intensities < 70% V̇O_2PEAK_ (Table 2). Aerobic exercise at 40–70% V̇O_2PEAK_ was feasible, as > 70% of participants completed > 75% of sessions at the prescribed intensity [ 34]. Aerobic exercise at 70–80% V̇O_2PEAK_ was infeasible as only 40% of participants completed > 75% of sessions at the prescribed intensity (Table 2).

The decline in compliance at 70% V̇O_2PEAK_ may reflect a transition between exercise performed below *vs.* above ventilatory threshold, which occurred at 70.6 ± 8.7% of V̇O_2PEAK_ pre-exercise training, thus suggesting that ventilatory threshold may represent a maximum tolerable dose that can be maintained for three 8-min exercise intervals in this cohort. However, 40% of participants reached the maximum gradient available on the treadmill when prescribed exercise at 70–80% V̇O_2PEAK_, suggesting that it may be possible for MGUS and SMM patients to achieve higher intensities than those shown in this study. The small sample size precluded differences in compliance to supervised aerobic exercise by intensity zone achieving statistical significance (*χ*^*2*^(3) = 4.024, *P =*.259). Supervised resistance exercise was progressive, but compliance to the prescribed repetition-maximum was poor (Table 2). The median home-based walk duration was 52 ± 11 min (prescription = 40 min), and intensity was RPE 11 ± 2, with the majority of home-based walks performed below the prescribed intensity of RPE 12–13/20 (Table 2).

No serious adverse events occurred. Grade one adverse events that were unrelated to the exercise programme– instead relating to pre-existing conditions– included illness/infection (*N* = 8 participants), knee pain (*N* = 2 participants), Achilles tendon pain (*N* = 1 participant), back pain (*N* = 1 participant), hand pain (*N* = 1 participant), gastric pain (*N* = 1 participant), and flare-up of psoriasis on feet (*N* = 1 participant). A grade one adverse event that was unlikely related to the exercise programme– due to the presence of other risk factors– was onset of symptoms consistent with plantar fasciitis (*N* = 1 participant). A grade one adverse event that was likely related to the exercise programme was muscle strain (*N* = 2 participants).

**Table 2:** Compliance to prescribed exercise intensity

Prescribed intensity	Actual intensity completed	Below target (%)		Compliant (%)	Above target (%)	Participants compliant for > 75% of sessions (%)
**Supervised aerobic exercise**						
40–50% V̇O_2PEAK_	48 ± 8	0 ± 0		100 ± 50	0 ± 25	93
50–60% V̇O_2PEAK_	56 ± 6	13 ± 20		86 ± 38	13 ± 14	80
60–70% V̇O_2PEAK_	64 ± 4	14 ± 34		83 ± 34	0 ± 0	73
70–80% V̇O_2PEAK_	70 ± 3	36 ± 53		67 ± 52	0 ± 0	40
**Supervised resistance exercise**						
15 repetitions	15 ± 0	0 ± 0		100 ± 0	0 ± 0	100
12 rep-max	14 ± 1	100 ± 25		0 ± 50	0 ± 0	7
10 rep-max	13 ± 2	100 ± 25		0 ± 25	0 ± 0	7
8 rep-max	11 ± 3	100 ± 8		0 ± 33	0 ± 0	7
**Home-based walking**						
RPE 12–13	11 ± 2	63 ± 67		38 ± 36	0 ± 17	13

Data are median ± IQR. Actual intensity calculated using heart rate measured by Polar H10 chest-based heart rate monitor for each session and baseline V̇O_2PEAK_ (supervised aerobic exercise), number of repetitions completed according to exercise record card completed by researcher (supervised resistance exercise), and RPE score reported by participants in exercise diaries (home-based walking). Compliant = within prescribed range; below target = lower intensity than prescribed range; above target = higher intensity than prescribed range. N.B. Columns do not sum to 100% due to medians calculated from data for *N* = 15 participants. V̇O_2PEAK_ = Peak oxygen uptake; Rep-max = Repetition-maximum; RPE = Rating of perceived exertion

### Disease biomarkers

**Fig. 2:**
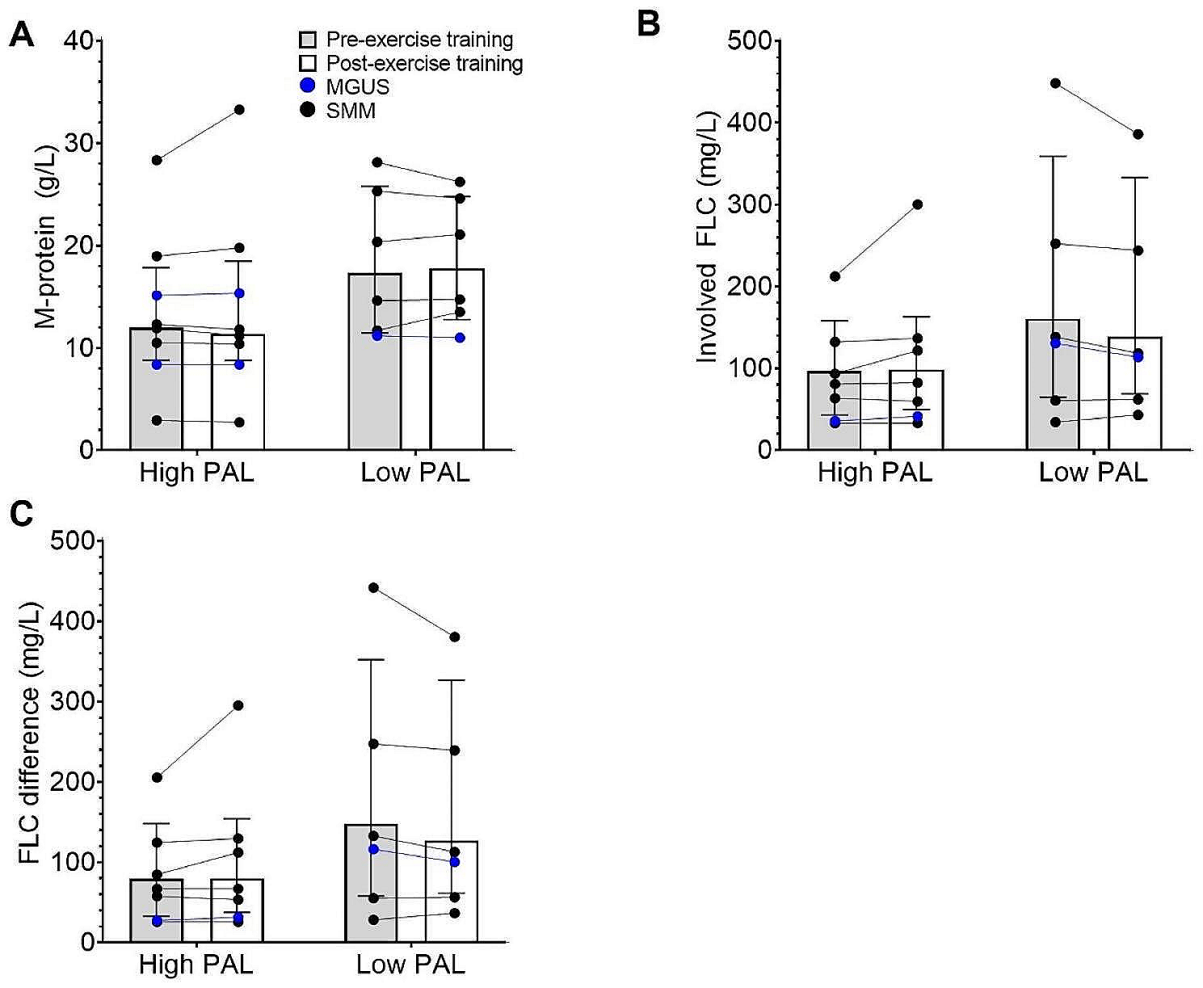
Pre- to post-exercise training changes to disease biomarkers in subgroups with a high *vs.* low baseline physical activity level. Pre- to post-exercise training changes to M-protein (**A**), involved FLC (**B**), and FLC difference (**C**) in subgroups of participants with a high baseline PAL (≥ 1.75) or low baseline PAL (< 1.75). Block bars show the median, and error bars show IQR. Lines joining circles show individual responses. PAL = Physical activity level; FLC = Free light chains

There was no change to serum M-protein (13.4 ± 8.6 *vs.* 14.0 ± 9.6 g/L, Δ 0.0 ± 1.0 g/L, *P* =.903, *N* = 14) or involved FLC (111.8 ± 92.6 *vs.* 135.8 ± 91.7 mg/L, Δ + 1.8 ± 16.8 mg/L, *P* =.839, *N =* 13) from pre- to post-exercise training. Furthermore, FLC difference was unchanged from pre- to post-exercise training (100.85 ± 92.13 *vs.* 119.34 ± 90.41 mg/L, Δ + 0.2 ± 15.6 mg/L, *P* =.946, *N =* 13). There was no statistically-significant effect of baseline physical activity level (e.g., high *vs.* low) on post-exercise training M-protein (*P* =.317), involved FLC (*P* =.133), or FLC difference (*P* =.140) when baseline differences to M-protein, involved FLC, and FLC difference, respectively, were accounted for (Fig. 2).

**Fig. 3:**
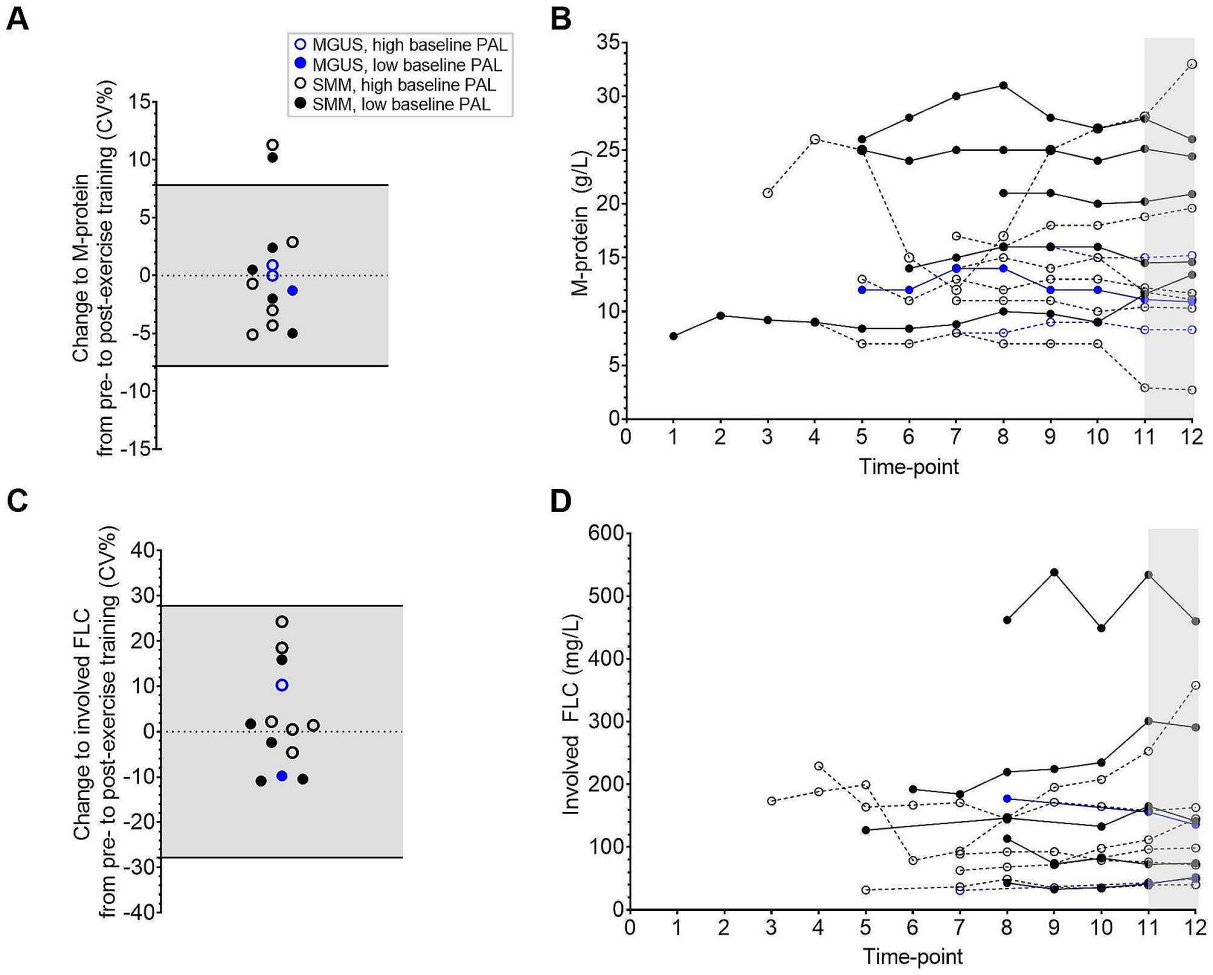
Individual changes to disease biomarkers of MGUS and SMM from pre- to post-exercise training. (**A**) Individual changes to M-protein shown in the context of physiological variation to M-protein during monitoring of stable disease (CV 7.8%) [35] shown in grey. (**B**) Individual changes to M-protein shown in the context of historical disease activity measurements accessed from medical records. Intervention period shown in grey. (**C**) Individual changes to involved FLC shown in the context of physiological variation to involved FLC during monitoring of stable disease (CV 27.8%) [35] shown in grey. (**D**) Individual changes to involved FLC shown in the context of historical disease activity measurements accessed from medical records. Intervention period shown in grey. High baseline physical activity level defined as PAL ≥ 1.75. Low baseline physical activity level defined as PAL < 1.75. In panel **B** and **D**, three-year disease history was available and, as the frequency of disease monitoring between participants varied, time-points were arbitrarily assigned 0–12 in order to align the intervention period in all participants. In panel **B** and **D**, where M-protein and involved FLC are shown to decrease in one participant between timepoints 5–7, it should be noted that this participant received treatment for prostate cancer during that period. FLC = Free light chains

Individual changes to M-protein and involved FLC from pre- to post-exercise training were generally within the physiological variation during monitoring of stable disease, with no reduction apparent, regardless of baseline physical activity level (Fig. 3A & C). Similarly, when displayed in the context of historical disease activity, pre- to post-exercise training changes appear to be consistent with usual fluctuations to M-protein (Fig. 3B) and involved FLC (Fig. 3D) during monitoring of stable disease in participants with both high and low physical activity levels at baseline.

The prevalence and severity of immunoparesis at baseline is shown in Table 1. There were no pre- to post-exercise training changes to polyclonal IgA (0.60 ± 0.81 *vs.* 0.56 ± 0.84 g/L, Δ + 0.01 ± 0.06 g/L, *P* =.663, *N* = 11), polyclonal IgG (6.39 ± 1.56 *vs.* 6.08 ± 1.44 g/L, Δ − 0.36 ± 0.64 g/L, *P* =.162, *N* = 4), or polyclonal IgM (0.37 ± 0.26 *vs.* 0.39 ± 0.23 g/L, Δ − 0.01 ± 0.04 g/L, *P* =.110, *N* = 15).

### Blood immunophenotype

#### CD4^+^ and CD8^+^ T cells

As shown in Table 3, there were no pre- to post-exercise training changes to CD3^+^, CD4^+^ or CD8^+^ T cell frequency, or the frequency of CD4^+^ or CD8^+^ naïve T cells (T_NA_, CD45RA^+^CD27^+^), central memory (T_CM_, CD45RA^−^CD27^+^), effector memory (T_EM_, CD45RA^−^CD27^−^) or terminally-differentiated effector memory cells re-expressing CD45RA^+^ (T_EMRA_, CD45RA^+^CD27^−^) (all *P* >.05, *d* < 0.2). There were no changes to CD57^+^CD4^+^, CD57^+^CD8^+^, KLRG1^+^CD4^+^, KLRG1^+^CD8^+^, PD1^+^CD4^+^, PD1^+^CD8^+^, or CTLA4^+^CD4^+^ T cells from pre- to post-exercise training (all *P* >.05, *d* < 0.2) (Table 3).

**Table 3:** Change to CD4^+^ and CD8^+^ T cell frequencies from pre- to post-exercise training

Cell frequency (cells/µL)	Pre-exercise training	Post-exercise training	Change	*P* statistic	Effect size
CD3^+^	1357 ± 424	1365 ± 459	115 ± 468	0.890	−
CD3^+^CD4^+^	985 ± 387	1118 ± 455	97 ± 381	0.720	−
CD4^+^ T_NA_	505 ± 294	501 ± 307	5 ± 108	0.897	0.0
CD4^+^ T_CM_	481 ± 245	501 ± 379	51 ± 194	0.789	0.1
CD4^+^ T_EM_	64 ± 36	68 ± 29	3 ± 22	0.934	0.0
CD4^+^ T_EMRA_	6 ± 6	6 ± 7	0 ± 2	0.354	0.0
CD57^+^CD4^+^	46 ± 32	48 ± 38	5 ± 16	0.421	−
KLRG1^+^CD4^+^	78 ± 84	78 ± 111	7 ± 27	0.703	0.0
PD1^+^CD4^+^	144 ± 84	139 ± 106	14 ± 37	0.454	−
CTLA4^+^CD4^+^	5 ± 3	5 ± 3	0 ± 3	0.625	0.1
CD3^+^CD8^+^	192 ± 112	196 ± 111	9 ± 55	0.581	0.1
CD8^+^ T_NA_	114 ± 72	103 ± 60	5 ± 26	0.934	−
CD8^+^ T_CM_	63 ± 47	51 ± 56	2 ± 17	0.600	−
CD8^+^ T_EM_	7 ± 6	7 ± 6	0 ± 2	0.600	−
CD8^+^ T_EMRA_	20 ± 26	16 ± 26	−2 ± 6	0.169	−
CD57^+^CD8^+^	45 ± 37	42 ± 29	−6 ± 11	0.188	−
KLRG1^+^CD8^+^	96 ± 83	92 ± 58	**−**4 ± 23	0.419	0.1
PD1^+^CD8^+^	37 ± 35	39 ± 36	−1 ± 8	0.679	−

Data are median ± IQR. All *N* = 15. Lymphocyte viability was 96.4% (range 92.7–99.1%). Cohen’s d effect sizes were calculated for normally distributed data only, as calculations for Cohen’s d are based on mean as the measure of central tendency, which does not appropriately reflect non-normal data distribution (indicated by– in the table). T_NA_ naïve T cell; T_CM_ = central memory T cell; T_EM_ = effector memory T cell; T_EMRA_ = terminally-differentiated effector memory cell re-expressing CD45RA^+^

### Blood inflammation

**Fig. 4:**
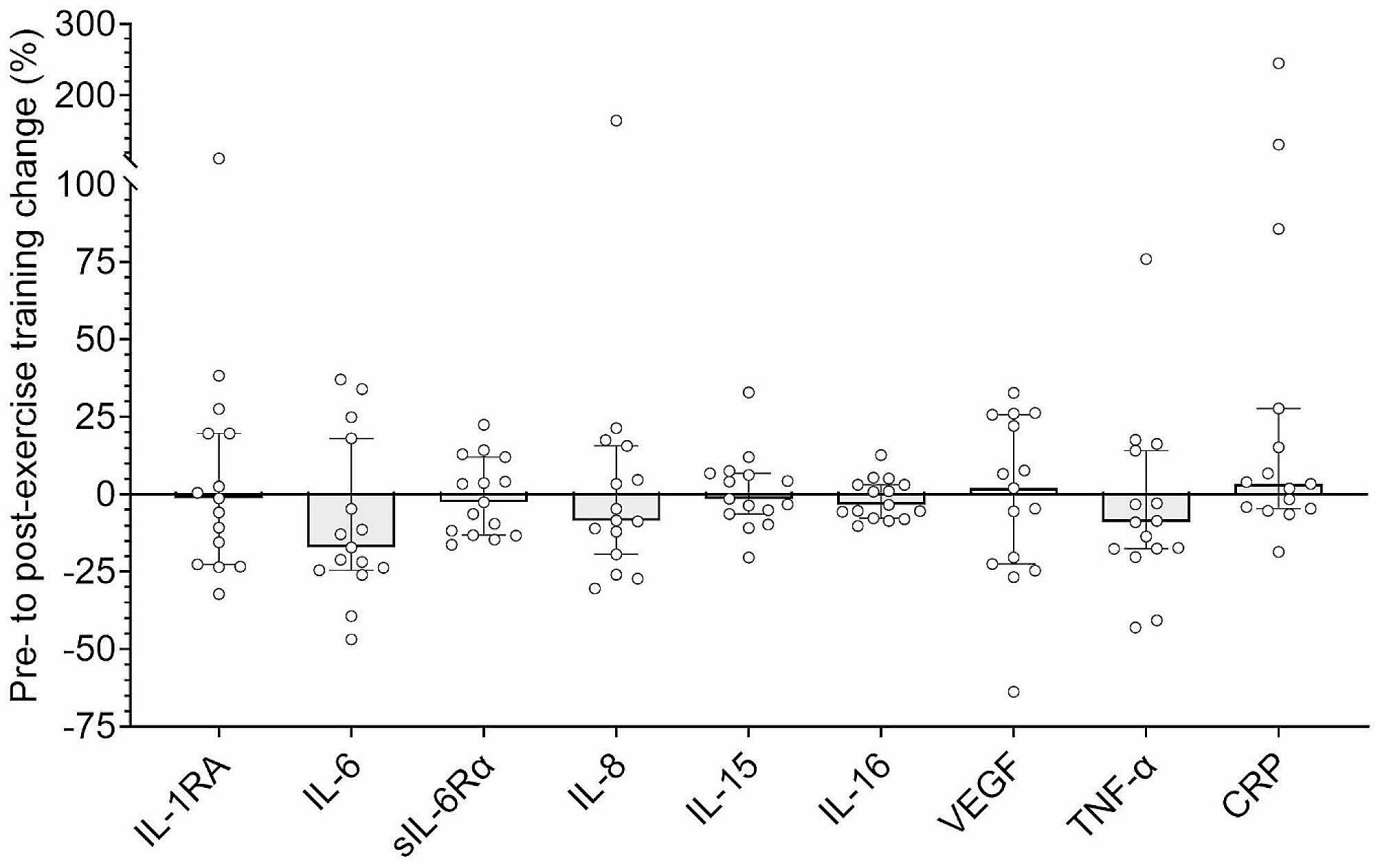
Percentage change to inflammatory biomarkers from pre- to post-exercise training Block bars are median percentage change from pre- to post-exercise training and error bars are IQR. Individual changes are shown in open circles. All *N* = 15, excluding IL-1RA (*N* = 10). All biomarkers are measured in plasma, except for CRP which was measured in serum. IL = Interleukin; RA = Receptor antagonist; CRP = C-reactive protein; VEGF = Vascular endothelial growth factor; sIL-6Rα = Soluble IL-6 receptor alpha; TNF-α = Tumour necrosis factor alpha

Plasma IL-1β, IL-4, IL-10, IL-12 and IL-17 A were undetectable (< 25% samples detected) and thus were not included in statistical analysis. There were no pre- to post-exercise training changes to plasma IL-1RA, IL-6, sIL-6Rα, IL-8, IL-15, IL-16, VEGF, TNF-α, or serum CRP (all *P* >.05, *d* < 0.2) (Fig. 4).

### Physiological measurements

#### Body composition

Waist circumference reduced by 2.8 ± 5.3 cm (*P* =.014, *d* = 0.2) from pre- to post-exercise training (Table 4). Body mass, BMI, and hip circumference were unchanged from pre- to post-exercise training (all *P* >.05) (Table 4). DEXA-derived measures of whole-body fat mass, lean soft-tissue mass, body fat percentage, bone mineral density, and bone mineral density T score were unchanged from pre- to post-exercise training (all *P* >.05, *N* = 14) (Table 4).

#### Resting cardiovascular measurements

There was a 3 ± 5 mmHg reduction (*P* =.033, *d* = 0.4) to diastolic blood pressure and a 3 ± 7 beats/min reduction (*P* =.014, *d* = 0.4) to resting heart rate, but no change to systolic blood pressure (*P* =.293) from pre- to post-exercise training (Table 4).

#### Cardiorespiratory fitness

The maximum work-rate achieved during the maximal treadmill exercise test increased by 3.8 ± 2.8 MET (*P* <.001, *d* = 1.4, *N* = 12) from pre- to post-exercise training (Table 4), but there was no change to V̇O_2PEAK_ or ventilatory threshold from pre- to post-exercise training (both *P* >.05, *N* = 12) (Table 4).

#### Functional fitness tests

Time to complete an 8ft up-and-go test reduced by 0.8 ± 0.9 s (*P* =.006, *d* = 0.8, *N* = 12) and the number of sit-to-stand repetitions performed in 30 s increased by 5 ± 5 repetitions (*P* =.002, *d* = 1.2, *N* = 12) from pre- to post-exercise training (Table 4). Grip strength increased by 2.0 ± 4.0 kg (*P* =.008, *N* = 14) from pre- to post-exercise training (Table 4). Upper limb flexibility was unchanged from pre- to post-exercise training (*P* >.05, *N* = 14), yet lower limb flexibility improved by 5.7 ± 7.7 cm (*P* =.044, *d* = 0.5, *N* = 13) (Table 4).

#### Quality of life

Energy and fatigue, and physical functioning sub-scores of the SF-36 increased by 10 ± 15% (*P* =.026, *d* = 0.5) and 5 ± 10% (*P* =.022), respectively, from pre- to post-exercise training (Table 4). Exercise training had no effect on physical role limitation, emotional role limitation, pain, and general health sub-scores of SF-36, plus FACIT-fatigue and PSQI sleep quality (all *P* >.05, *d* < 0.2) (Table 4). Small effect sizes were detected for pre- to post-exercise training increases to life satisfaction (Δ + 1 ± 6 points, *P* =.202, *d* = 0.3) and emotional wellbeing (Δ + 4 ± 12%, *P* =.285, *d* = 0.2), although these changes were not statistically significant (Table 4).

**Table 4:** Pre- to post-exercise training changes to body composition, resting cardiovascular measurements, cardiorespiratory and functional fitness, and quality of life

Variable		Pre-exercise training	Post-exercise training	Pre- to post-exercise training change		*P* statistic	Effect size
**Body composition**							
Body mass (kg)		80.4 ± 31.4	80.6 ± 28.0	−0.5 ± 2.4		0.871	0.0
Body mass index (kg/m^2^)		27.3 ± 8.7	27.1 ± 9.0	−0.2 ± 0.8		0.759	0.0
Waist circumference (cm)		98.2 ± 29.3	93.8 ± 20.9	−2.8 ± 5.3		**0.014**	0.2
Hip circumference (cm)		103.9 ± 15.6	101.6 ± 16.6	−0.7 ± 3.0		0.070	0.1
Body fat percentage (%, *N* = 14)		30.4 ± 10.0	32.1 ± 10.6	0.2 ± 1.6		0.924	0.0
Fat mass (kg, *N* = 14)		20.9 ± 18.3	21.9 ± 19.4	0.2 ± 2.2		0.952	−
Lean soft-tissue mass (kg, *N* = 14)		58.8 ± 17.9	58.6 ± 17.8	−0.1 ± 1.0		0.818	0.0
Bone mineral density (g/cm^2^, *N* = 14)		1.18 ± 0.22	1.20 ± 0.22	0.00 ± 0.02		0.866	0.0
Bone mineral density T score (*N* = 14)		0.05 ± 2.60	0.15 ± 2.40	−0.05 ± 0.20		0.640	0.0
**Resting cardiovascular measurements**							
Systolic blood pressure (mmHg)		142 ± 18	132 ± 17	−4 ± 14		0.293	0.1
Diastolic blood pressure (mmHg)		86 ± 11	81 ± 10	−3 ± 5		**0.033**	0.4
Resting heart rate (beats/min)		67 ± 6	65 ± 7	−3 ± 7		**0.014**	0.4
**Cardiorespiratory and functional fitness**							
V̇O_2PEAK_ (mL/kg/min, *N* = 12)		26.2 ± 4.5	26.0 ± 4.5	0.1 ± 2.7		0.601	0.1
Peak work-rate (METs, *N* = 12)		9.9 ± 3.1	14.9 ± 4.0	3.8 ± 2.8		**0.001**	1.4
Ventilatory threshold (mL/kg/min, *N* = 12)		18.1 ± 2.9	17.8 ± 3.1	−1.3 ± 2.1		0.569	0.1
8ft up-and-go (s, *N* = 12)		5.6 ± 1.3	4.6 ± 1.0	−0.8 ± 0.9		**0.006**	0.8
Sit-to-stand (number in 30s, *N* = 12)		13 ± 3	17 ± 4	5 ± 5		**0.002**	1.2
Grip strength (kg, *N* = 14)		30.1 ± 12.9	34.5 ± 15.2	2.0 ± 4.0		**0.008**	−
Upper limb flexibility (cm, *N* = 14)		−3.4 ± 9.8	−3.6 ± 12.7	0.8 ± 1.5		0.101	−
Lower limb flexibility (cm, *N* = 13)		7.8 ± 15.0	11.9 ± 7.6	5.7 ± 7.7		**0.044**	0.5
**Quality of life**							
Satisfaction with life scale		24 ± 11	25 ± 9	1 ± 6		0.202	0.3
FACIT-fatigue scale		5 ± 9	2 ± 11	0 ± 5		0.640	0.1
PSQI		6 ± 5	5 ± 5	0 ± 4		0.900	0.0
SF-36 Physical function (%)		90 ± 30	95 ± 18	5 ± 10		**0.022**	−
SF-36 Physical role limitation (%)		100 ± 13	100 ± 0	0 ± 13		0.594	−
SF-36 Emotional role limitation (%)		100 ± 0	100 ± 0	0 ± 0		0.999	−
SF-36 Energy and fatigue (%)		65 ± 38	75 ± 23	10 ± 15		**0.026**	0.5
SF-36 Emotional wellbeing (%)		84 ± 22	84 ± 22	4 ± 12		0.285	0.2
SF-36 Social functioning (%)		88 ± 13	100 ± 19	0 ± 13		0.364	0.2
SF-36 Pain (%)		80 ± 23	90 ± 26	10 ± 15		0.272	−
SF-36 General health (%)		55 ± 15	55 ± 28	0 ± 8		0.342	0.1

Data are median ± IQR. *N* = 15 unless otherwise stated. Cohen’s d effect sizes were calculated for normally distributed data only, as calculations for Cohen’s d are based on mean as the measure of central tendency, which does not appropriately reflect non-normal data distribution (indicated by– in the table). A negative score on the back-scratch test indicates a gap between fingers and on the sit-and-reach test indicates not reaching the toes. A positive score on the back-scratch test indicates overlapping fingers and, on the sit-and-reach test, indicates reaching past the toes. V̇O_2PEAK_ = Peak oxygen uptake; METs = Metabolic equivalent of task; FACIT = Functional assessment of chronic illness therapy questionnaire; PSQI = Pittsburgh sleep quality index; SF-36 = 36-item short form questionnaire v1.0.

## Discussion

The principal finding of this pilot study was that short-term progressive exercise training was feasible and safe for patients with MGUS/SMM who enrolled in this trial and passed screening. Indeed, there were no serious adverse events, retention was high, adherence to the intervention was excellent, and compliance to the exercise prescription was high overall. However, the low rate of participant uptake indicates that a revised trial design should be implemented in a future RCT to attract more participants.

Prevalent barriers of ‘lack of time’ and ‘travel burden’ were reported by patients who declined involvement in the trial. The exercise programme was largely delivered via supervised exercise sessions within the hospital, with the aim of maximising compliance to specific exercise intensity prescriptions and providing instant access to medical assistance if required. However, this design required a total of 35 hospital visits which reduced accessibility of the trial where the catchment area of the hospital extends > 30 miles. In a future RCT, virtually-supervised exercise sessions could be trialled to improve uptake, as such an approach has been shown to be feasible in patients with MM, a more advanced disease stage than MGUS/SMM [36, 37]. Such modifications to the trial design warrant further evaluations of feasibility and safety across a broader range of MGUS/SMM patients.

The exercise protocol used herein was purposefully designed to evaluate the feasibility of vigorous intensity aerobic exercise in MGUS/SMM. This is firstly because a prior case study linking reductions to SMM disease activity with exercise training included high-intensity exercise modalities [9], but principally because it is commonly proposed that acute bouts of exercise augment cancer immunosurveillance and elimination in peripheral tissues, in an intensity-dependent manner [14–16]. We found that vigorous intensity aerobic exercise was feasible in the MGUS/SMM patients enrolled in this study, but only at an intensity marginally above moderate intensity. Indeed, when the exercise prescription progressed from moderate to vigorous intensity (at 60–70% V̇O_2PEAK_), the vast majority (> 80%) of treadmill walks were performed at the target (vigorous) intensity. However, our investigation identified that 70% V̇O_2PEAK_ may represent a maximum tolerated intensity when performed for three, 8-min intervals in MGUS/SMM, as compliance to 70–80% V̇O_2PEAK_ was considerably lower (~ 60%) and thus deemed infeasible at that intensity. However, despite poorer compliance at 70–80% V̇O_2PEAK_, the average intensity achieved for all participants at this prescription was 70% V̇O_2PEAK_, indicating that vigorous intensity exercise was still performed. Thus, overall, participants performed vigorous intensity exercise twice-weekly for ten weeks of the intervention.

A key secondary finding of this pilot study was that short-term progressive exercise training did not induce statistically significant or clinically meaningful changes (i.e., reductions) to biomarkers of MGUS and SMM disease activity. Indeed, changes to disease biomarkers did not exceed physiological variation observed during monitoring of stable disease [35] and did not meet the minimum criteria for response to anti-MM therapy of a 25% reduction from baseline [38]. The lack of change to involved FLC– which have a shorter half-life of 2–6 h compared with 5–23 days for intact immunoglobulins [39] and thus provide near real-time monitoring of changes to disease activity– from pre- to post-exercise training mitigates any doubt that the absence of a reduction to M-protein following 16 weeks of exercise training is due to its relatively long half-life. Thus, the present study shows that a 16-week period of exercise training neither measurably improves nor worsens MGUS and SMM disease activity. Our findings contradict those of a prior case study whereby a young, former elite athlete diagnosed with SMM had a decline in M-protein of 27% during a supervised, multi-modal exercise training programme, albeit this occurred over a three-year time period [ 9].

The findings of this investigation align with those of a recent study in watch-and-wait chronic lymphocytic leukaemia, where there was no reduction to tumour cells reported in those who completed a 12-week high-intensity exercise training programme [34]. Watch-and-wait chronic lymphocytic leukaemia has similarities to MGUS/SMM, as it represents a treatment-naïve B cell lineage cancer which is clinically monitored via blood testing, and it is a disease that mostly affects older adults. Similar results have also been observed in the related model of active surveillance for localised prostate cancer [40–42]. Indeed, in aerobic exercise intervention trials spanning 12 weeks to 12 months during active surveillance for prostate cancer, prostate-specific antigen (PSA) was unchanged [42] or reduced modestly (CV 2.9–6.6%) [40, 41]– within the physiological variation for PSA (CV ~ 7%) [43]– in the exercise group. The clinically-insignificant reduction to PSA in these studies - and the findings from our study - suggests that existing precursor disease cannot be unilaterally eliminated - e.g., via exercise-induced immunosurveillance - arising from short-term exercise training. Additionally, we found that M-protein and FLC were unchanged by short-term exercise training even in participants with low physical activity levels prior to commencement of this trial. It should be noted, however, that subgroup analysis according to baseline physical activity level was not planned *a priori* and includes only a small number of participants within each subgroup. 

In this study, we did not observe any changes to blood immunophenotype in the participants enrolled in the exercise intervention. Given that the anti-cancer properties of exercise are likely derived over a longer time horizon, this is perhaps an unsurprising result. Indeed, it has been proposed that exercise elicits anti-cancer effects by preserving immune function (e.g., diversity, persistence) against cancer clones that have mutated to become immunogenic [13]. It is tempting to suggest that this is demonstrated in Fig. 3, where there appears to be a pattern for lower disease activity in participants with a high physical activity level prior to commencement of the trial.

This study cannot exclude the possibility that exercise augmented immunosurveillance against recently mutated immunogenic cells, which may have been identified in this study were a control group included. For example, in watch-and-wait chronic lymphocytic leukaemia, there was a greater increase in disease activity– measured via enumeration of tumour cells in blood– in a non-exercise control group, compared to those completing an exercise training programme [34]. This supports the idea that exercise augments anti-tumour responses against immunogenic cell clones, but not clones lacking immunogenicity. However, it does not confirm that this response arises as a result of acute-exercise induced immunosurveillance, and only that exercise training elicited this outcome. Regardless, longitudinal studies are required to evaluate the longer-term effects of regular exercise on disease progression from cancer precursors (e.g., MGUS and SMM) to symptomatic cancer (e.g., MM). 

A strength of this study is the breadth and depth of secondary outcome measures. Firstly, we found that body fat percentage estimated by DEXA and BMI were unchanged from pre- to post-exercise training. This likely explains why inflammatory mediators were also unchanged in the present study, as adipose tissue– particularly in older age– is a key contributor to systemic inflammation [44]. Nevertheless, the largest meta-analysis to date revealed that associations between self-reported physical activity level and clinical cancer risk withstood adjustment for BMI for nearly all cancer sites, including MM [10]. As such, the lack of change to body composition and associated inflammation herein does not undermine the proposition that MGUS and SMM disease activity cannot be reversed by 16 weeks of exercise, as it is likely that mechanisms independent of obesity are involved in the association between physical activity and cancer risk.

In the present study, we also observed no change to lean soft-tissue mass estimated by DEXA, which may be explained by immune dysfunction in MGUS and SMM. Indeed, the repair of muscle damage induced by exercise, which results in muscle hypertrophy, is dependent on a coordinated immune response [45] and immunoparesis– an indicator of immunosuppression– was present in 72% of participants in the present trial. Alternatively, a more likely explanation is that the exercise prescription was insufficient to induce muscle hypertrophy. Indeed, despite resistance exercise progressively increasing in load, compliance to repetition-maximum targets was consistently poor in this study. Exercise prescriptions to induce muscle hypertrophy are relevant to MGUS and SMM, as increases to muscle-derived cytokines in physically active individuals may be involved in maintaining immune competence against immunogenic cancer cells to reduce clinical cancer risk [13]. As such, the lack of change to blood immunophenotype may be due to lack of muscle hypertrophy following the 16-week exercise programme. Future studies should include measurements of immune cells in the tumour microenvironment, which are likely to yield more insightful information regarding the anti-tumour immunological effects of exercise in comparison to measurements of immune cell phenotypes in blood [13].

With regards to measurements of fitness, the maximum work-rate attained during CPET increased from pre- to post-exercise training, indicating an improvement in efficiency and exercise tolerance. Additionally, we detected improvements in resting heart rate and diastolic blood pressure suggestive of improved resting cardiovascular function following 16 weeks of progressive exercise training. We also observed improvements to functional fitness (e.g., sit-to-stand 30, 8ft up-and-go) from pre- to post-exercise training in the present study. In contrast, we found that V̇O_2PEAK_ was unchanged from pre- to post-exercise training, which has also been shown previously following exercise training in MM [19, 20]. Disease pathophysiology of MGUS, SMM, and MM may preclude improvements to V̇O_2PEAK_, as the oxygen carrying capacity of blood is a limiting factor [46] and the expansion of clonal plasma cells compromises the production of erythrocytes in the bone marrow [47] which may affect oxygen transport. Indeed, erythrocyte count and haemoglobin were unchanged from pre- to post-exercise training in the present study (data not shown). Another potential explanation for the lack of change to V̇O_2PEAK_ from pre- to post-exercise training is the higher prevalence of sub-maximal tests at follow-up compared to baseline. However, it cannot be discounted that the exercise prescription may have been insufficient to increase V̇O_2PEAK_.

Global quality of life scores crossed a threshold from ‘average’ to ‘high’ life satisfaction from pre- to post-exercise training in our study, suggesting a meaningful improvement [48]. Furthermore, improvements to physical functioning and energy/fatigue domains of health-related quality of life measured via SF-36 occurred from pre- to post-exercise training. However, fatigue measured via FACIT fatigue scale, plus PQSI sleep quality and other domains of SF-36 health-related quality of life, were unchanged. To the authors’ knowledge, this is the first study to report improvements to aspects of quality of life in MGUS/SMM patients following exercise training. This may be of clinical significance, as individuals with MGUS and SMM report similar levels of mental health-related quality of life, psychological distress, and anxiety to MM patients [49], and quality of life scores predict overall survival in newly diagnosed MM [50].

It should be considered that despite using a ‘within-patient control’ approach using historical disease activity data, the single-arm design of this pilot study does not capture changes to disease activity that occur– in the absence of an exercise training programme– over a 16-week period in participants during ‘watchful waiting’ usual care. Indeed, as anti-cancer therapy is not advocated in MGUS and SMM [8] disease activity biomarkers are expected to steadily increase over time. Indeed, it is plausible that M-protein and FLC could increase within a period of 16 weeks, as guidelines recommend monitoring newly-diagnosed SMM after 8–12 weeks initially, increasing to 16–24 weeks if stable [8]. Furthermore, the single-arm design precludes improvements to general health outcomes being definitively attributed to the exercise programme. In addition, while this pilot study captured a range of participants of different ages and sex– which greatly expands the available evidence from one patient with SMM [9]– a larger sample is required to include diversity in ethnicity and clinical status. A larger sample size is also required for meaningful interpretation of pre-post statistical analyses, which are underpowered in the present pilot study. Additionally, the exercise programme was designed to incorporate progressively higher intensity exercise, which may not be suitable for all MGUS/SMM patients, as treadmill walking for 30 min at 70–80% V̇O_2PEAK_ was shown not to be feasible in this study. Lastly, the resistance exercise prescription could be optimised for muscle hypertrophy in conjunction with appropriate nutritional guidance.

## Conclusions

To conclude, this single-armed pilot study found that a 16-week progressive exercise training programme was safe and feasible in MGUS/SMM patients enrolled in this trial, and did not improve nor worsen MGUS/SMM disease activity. As such, exercise training could be recommended to MGUS/SMM patients to attain other health benefits identified herein for the first time, including improvements to blood pressure, exercise tolerance, functional fitness, and aspects of quality of life. Future studies should explore the effects of exercise over a longer time horizon (e.g., 12 + months), and measure time-to-progression from MGUS/SMM to MM.

### Electronic supplementary material

Below is the link to the electronic supplementary material.

Supplementary Material 1

## Data Availability

The datasets used and/or analysed during the current study are available from the corresponding author on reasonable request.

## Abbreviations

ACSM: American College of Sports Medicine
BMI: Body Mass Index
CERT: Consensus on Exercise Reporting Template
CPET: Cardiopulmonary Exercise Test
CRP: C-Reactive Protein
CV: Coefficient of Variation
DMSO: Dimethyl Sulfoxide
DEXA: Dual-Energy X-Ray Absorptiometry
ECG: Electrocardiogram
ECOG: Eastern Co-operative Oncology Group
FACIT: Functional Assessment of Chronic Illness Therapy
FCS: Foetal Calf Serum
FLC: Free Light Chains
IL: Interleukin
MGUS: Monoclonal Gammopathy of Undetermined Significance
MM: Multiple Myeloma
M-protein: Monoclonal Protein
NK: Natural Killer
NHS: National Health Service
PAL: Physical Activity Level
PBMC: Peripheral Blood Mononuclear Cells
PBS: Phosphate-Buffered Saline
RPE: Rating of Perceived Exertion
PQSI: Pittsburgh Sleep Quality Index
RPMI: Roswell Park Memorial Institute
SF-36: 36-Item Short Form Questionnaire
SMM: Smouldering Multiple Myeloma
VEGF: Vascular Endothelial Growth Factor
V̇O_2PEAK_: Peak Oxygen Uptake
