# Supplementary material for: The effects of short-term, progressive exercise training on disease activity in smouldering multiple myeloma and monoclonal gammopathy of undetermined significance: a single-arm pilot study
Source: BMC Cancer. 2024 Feb 5;24:174. doi: 10.1186/s12885-024-11817-6 (PMC10840198; doi:10.1186/s12885-024-11817-6)
Supplement: Supplementary file 1 — Supplementary Material 1 [file 12885_2024_11817_MOESM1_ESM.pdf]

**Supplemental Figure 1.** Flow cytometry gating strategy for identifying T cell populations.

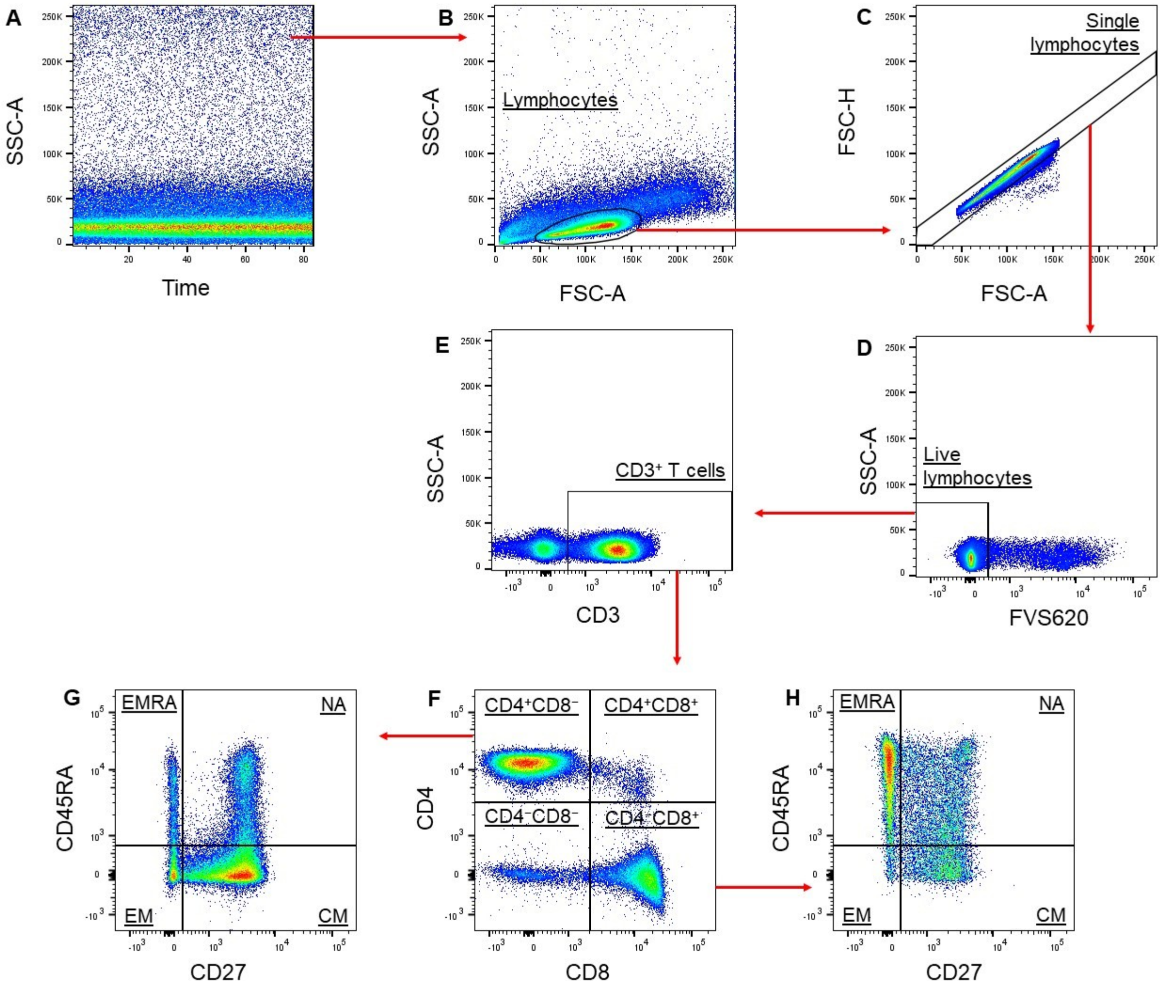


*(A) Time gating quality control performed to exclude cells acquired during periods of unstable fluidics; (B) Gating on SSC-A and FSC-A to identify lymphocytes according to morphology; (C) Gating on FSC-H and FSC-A to exclude doublets; (D) Gating on live-dead stain to identify living lymphocytes; (E) Gating on CD3⁺ T cells; (F) Quadrant gating of CD4 and CD8 to identify CD4⁺ and CD8⁺ T cells, plus double-positive and double-negative T cells; (G) Quadrant gating of memory phenotypes within CD4⁺ T cells to identify $T_{NA}$, $T_{CM}$ and $T_{EM}$ and $T_{EMRA}$; (H) Quadrant gating of memory phenotypes within CD8⁺ T cells to identify $T_{NA}$, $T_{CM}$ and $T_{EM}$ and $T_{EMRA}$. Expression of CTLA4, PD1, CD57, and KLRG1 was examined within total CD4⁺ T cell and CD8⁺ T cell populations. $T_{NA}$ = Naïve T cell; $T_{CM}$ = Central memory T cell; $T_{EM}$ = Effector memory T cell; $T_{EMRA}$ = Effector memory T cell re-expressing CD45RA⁺; CTLA4 = Cytotoxic T-lymphocyte associated protein 4; PD1 = Programmed cell death protein 1; KRLG1 = Killer cell lectin like receptor G1.*

**Supplemental Figure 2.** Schematic diagram showing the structure of supervised and home-based exercise sessions, in the context of the 16-week exercise programme.

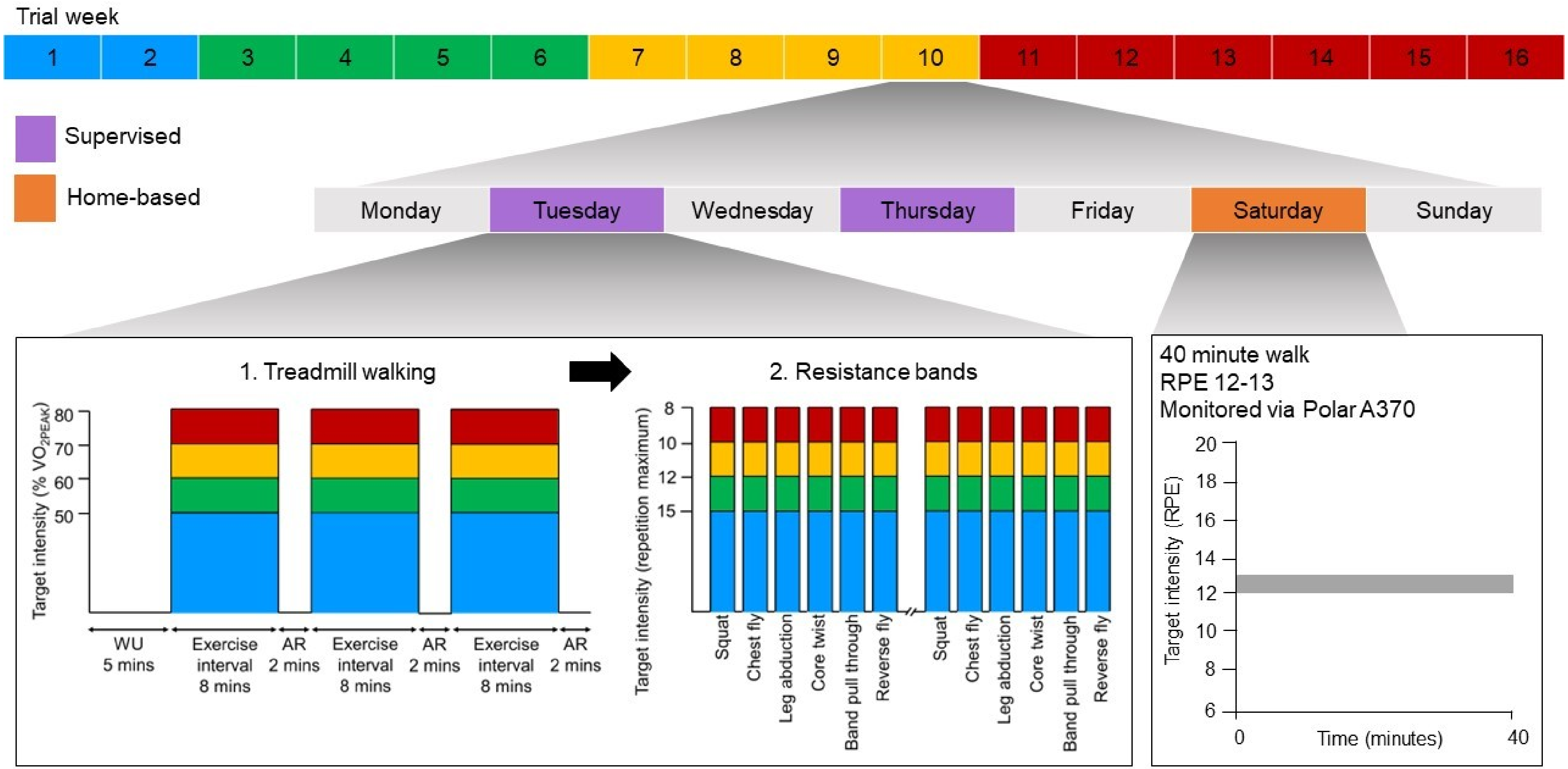


*Each trial week comprised two supervised and one home-based exercise session. Colour-coding is used to link the trial week to the prescribed exercise intensities: Blue = weeks 1-2, 40-50% $\dot{V}O_{2PEAK}$, 15 repetitions; Green = weeks 3-6, 50-60% $\dot{V}O_{2PEAK}$, 12 repetition-maximum; Yellow = weeks 7-10, 60-70% $\dot{V}O_{2PEAK}$, 10 repetition-maximum; Red = weeks 11-16, 70-80% $\dot{V}O_{2PEAK}$, 8 repetition-maximum. Supervised sessions commenced with a five-minute warm-up, followed by 30 minutes of treadmill walking (8 minutes work:2 minutes active recovery). Next, two sets of six standardised resistance exercises were performed, unless participants experienced pain in given movement patterns, where substitutions were offered, e.g., reverse fly → low row, core twist → side bend, squat → sit-to-stand. Home-based exercise was a 40-minute walk at moderate intensity. The total prescribed weekly volume of aerobic exercise was 100 minutes at moderate intensity in weeks 1-6 and 100 minutes at moderate-vigorous intensity in weeks 7-16. WU = Warm-up; AR = Active recovery; $\dot{V}O_{2PEAK}$ = Peak oxygen uptake; RPE = Rating of perceived exertion.*

**Supplemental Table 1.** Consensus on Exercise Reporting Template (CERT) reporting of exercise programme.

| Section/topic | Item # | Checklist item | Location | |
|---|---|---|---|---|
| | | | Primary paper (page, table, appendix) | Other (paper or protocol, website URL) |
| WHAT: materials | 1 | Detailed description of the type of exercise equipment (e.g., weights, exercise equipment such as machines, treadmills, bicycle ergometer etc) | Page 6-7 | |
| WHO: provider | 2 | Detailed description of the qualifications, teaching/supervising expertise, and/or training undertaken by the exercise instructor | Page 6 | |
| HOW: delivery | 3 | Describe whether the exercises are performed individually or in a group | Page 6 | |
| | 4 | Describe whether the exercises are supervised or unsupervised and how they are delivered | Page 6-7 | |
| | 5 | Detailed description of how adherence to exercise is measured and reported | Page 7 | |
| | 6 | Detailed description of motivational strategies | N/A | |
| | 7a | Detailed description of the decision rule(s) for determining the exercise progression | Page 7 | |
| | 7b | Detailed description of how the exercise programme was progressed | Page 7 | |
| | 8 | Detailed description of each exercise to enable replication (e.g., photographs, illustrations, videos etc) | N/A | |
| | 9 | Detailed description of any home programme components (e.g., other exercises, stretching etc) | Page 7 | |
| | 10 | Describe whether there are any non-exercise components (e.g., education, cognitive behavioural therapy, massage etc) | N/A | |
| | 11 | Describe the number and type of adverse events that occurred during exercise | Page 8 | |
| WHERE: location | 12 | Describe the setting in which the exercises are performed | Page 6-7 | |
| WHEN, HOW MUCH: dosage | 13 | Detailed description of the exercise intervention including, but not limited to, number of exercise repetitions/sets/sessions, session duration, intervention/programme duration etc | Page 7 | |
| TAILORING: what, how | 14a | Describe whether the exercises are generic (one size fits all) or tailored to the individual | Page 7 | |
| | 14b | Detailed description of how exercises are tailored to the individual | Suppl. Fig 2 | |
| | 15 | Describe the decision rule for determining the stating level at which people commence an exercise programme (such as beginner, intermediate, advanced etc) | N/A | |
| HOW WELL: planned, actual | 16a | Describe how adherence or fidelity to the exercise intervention is assessed/measured | Page 7 | |
| | 16b | Describe the extent to which the intervention was delivered as planned. | Page 8 | |
